# Artificial intelligence–assisted augmented reality robotic lung surgery: Navigating the future of thoracic surgery

**DOI:** 10.1016/j.xjtc.2024.04.011

**Published:** 2024-05-03

**Authors:** Amir H. Sadeghi, Quinten Mank, Alper S. Tuzcu, Jasper Hofman, Sabrina Siregar, Alexander Maat, Alexandre Mottrie, Jolanda Kluin, Pieter De Backer

**Affiliations:** a Department of Cardiothoracic Surgery, Erasmus Medical Center, Rotterdam, The Netherlands; b Department of Cardiothoracic Surgery, University Medical Center Utrecht, Utrecht, The Netherlands; c MedicalVR, Nieuw-Vennep, The Netherlands; d Orsi Academy, Melle, Belgium

**Figure:**
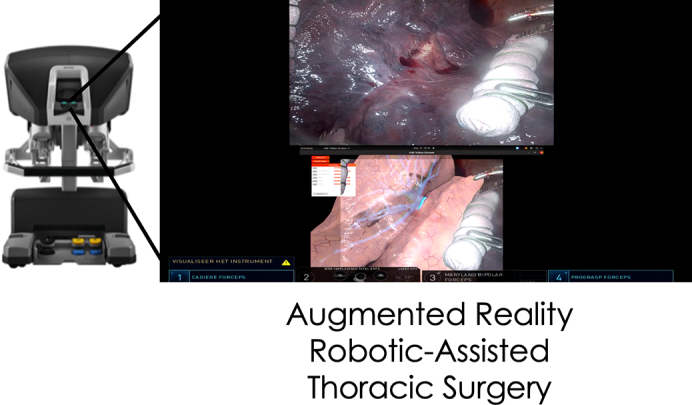
Real-time AI-assisted augmented reality view during robotic-assisted thoracic surgery.

**Central Message**

We investigated the technological feasibility of an AI-based approach to enable augmented reality (registration/dynamic tracking) robotic lung surgery with deformable and interactive 3D-models.

In recent years, thoracic surgery has undergone a transformative shift, propelled by technological advancements such as robotic-assisted thoracic surgery (RATS), artificial intelligence (AI), and extended reality.^1^ RATS has gained global popularity, offering innovative tools like robotic stapling, fluorescence imaging, and the integration of 3-dimensional (3D) reconstructions in the console. However, challenges persist, including a lack of tactile feedback, constrained visual access because of the proximity of robotic instruments to target tissues, and a significant learning curve.^2^ In addition, preoperative (3D) computed tomography scans do not accurately represent the anatomical deformations in the lung's shape during RATS.^3^ The integration of AI/extended reality has emerged as a potential solution, providing real-time anatomical updates and ensuring that the surgeon has an accurate and unobscured representation of the patient's 3D anatomy.^4^^,^^5^ Integrating these technologies in RATS anatomical lung resections will hopefully enhance precision, safety, surgeon satisfaction, and surgical outcomes of lung cancer patients. This article explores the feasibility of a technological milestone by combining AI, augmented reality (AR), and pulmonary robotic surgery, showcasing the first-in-human AR robotic lobectomy.

## Methods

### Technology

In collaboration with MedicalVR, we have developed PulmoVR's (MedicalVR) deep learning (DL)-based segmentation method, which automatically generates 3D reconstructions from the computed tomography scan, focusing on pulmonary artery, vein, lobes, and airways. In order to create these models, 126 computed tomography scans from patients who underwent lung resection at Erasmus MC were collected over years. To train a DL model, the pulmonary arteries and veins were manually annotated and subsequently used for the development of the AI-based algorithm.

**Figure E1:**
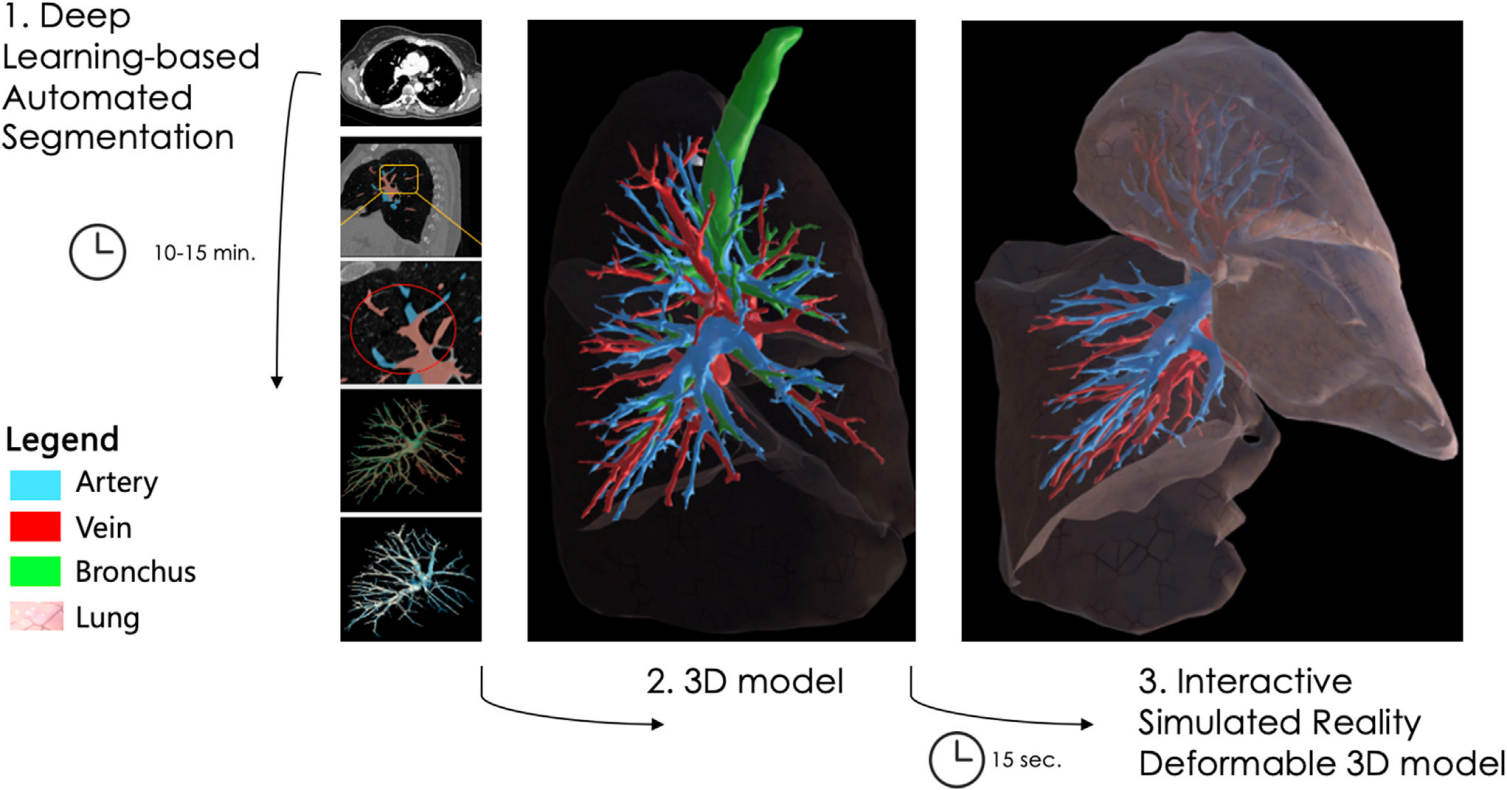
Automated approach to facilitate interactive simulated reality 3-dimensional (*3D*) lung modeling. First, the computed tomography Digital Imaging and Communications in Medicine files are used and processed by a deep learning–based algorithm to create a 3D lung model. After that, these models can be automatically transformed into interactive simulated reality models using realistic finite element method simulations which are integrated in the PulmoVR software platform.

These segmented images are then transformed into interactive and dynamic simulated realities (iSRs; interactive 3D models that have the ability to closely simulate the reality in terms of lung deformation) through finite element method simulations (virtual computer-based simulations, enabled by applying physics-based principles) creating deformable virtual 3D models (Figure E1). Subsequently, users (eg, the surgeon) choose the segmented images created with the aforementioned DL model. The software automatically selects a physics template, defining biomechanical properties. iSR allows the user to deform (with a mouse) the models in real time with simple motion-based input, which then adapt boundary conditions of the finite element method simulation. This means that the user (bedside technician) can deform the model manually according to his/her interpretation on the extent of compression/deformation that is carried out by the console surgeon. However, it is not possible yet to deform the virtual models automatically.

**Figure 1:**
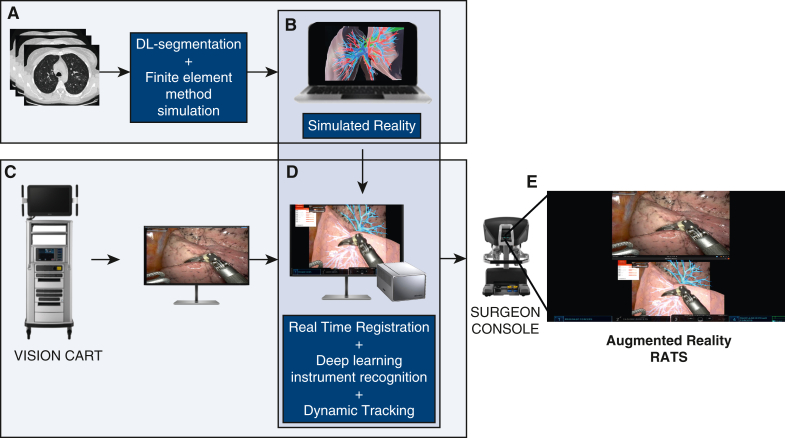
Artificial intelligence and augmented reality-guided robotic thoracic surgery setup. A, After obtaining computed tomography Digital Imaging and Communications in Medicine files, a deep learning (*DL*)-based anatomical segmentation is performed to create patient-specific static 3-dimensional (*3D*) lung models, which can subsequently be transformed into interactive simulated reality models using realistic finite element method simulations (B). These models can be deformed on a laptop (or desktop) by a user (eg, by a surgeon, clinical technician, etc), who is also receiving a live-stream of the thoracoscopic video, extracted from the robotic vision cart (C). On a second high-performance computer (NVIDIA Holoscan), the manual AR registration (merging of iSR with thoracoscopic video) can be performed where a DL-based algorithm detects and filters out the robotic instruments to create a realistic superimposition of the iSR model on top of the corresponding anatomy, where the instruments can still be seen (D). Subsequently, these video frames are displayed on the robotic console via Intuitive Surgical's Tilepro input (E). *iSR*, Interactive simulated reality.

For real-time instrument deocclusion, a DL algorithm segments nonorganic items.^5^ The technology consists of a robust real-time binary segmentation pipeline for nonorganic items, where the segmented instruments are rendered on top of the patient-specific 3D model to mitigate instrument deocclusion and add a sense of depth to the augmented reality. The dataset for binary segmentation comprises 31,812 images in which nonorganic items were outlined using the SuperAnnotate annotation platform. These nonorganic items cover a variety of categories such as robotic and laparoscopic instruments, needles, wires, clips, vessel loops, bulldogs, gauzes, and more. The selection of images was uniformly drawn from 100 full-length robotic-assisted partial nephrectomy procedures. The segmented instruments overlay the iSR 3D-model, enhancing AR with depth perception. Developed with NVIDIA Holoscan SDK (leveraging the Clara AGX developer kit), the intraoperative AI and AR application captures endoscopic video processed by a Deltacast capture card. The output feeds into the Tilepro input for the surgical console and a bedside monitor simultaneously, allowing real-time AR views for the surgeon and real-time model alignment by a bedside technician (Figure 1). This will enable a real-time colored overlay of a dynamic 3D model, mirroring the lung as it is manipulated by the surgeon.

### Feasibility

Technological feasibility of this platform was assessed during 2 consecutive surgical cases of a right and left lower lobectomy. Patient's informed consent and ethical board review (MEC-2023-0080/0397; date of approval: February 26, 2023, and August 31, 2023) were obtained. Feasibility was defined as a successful and anatomically correct (as judged by the surgeon) real-time registration of the patient-specific dynamic deformable lung model onto the corresponding anatomy during 3 surgical phases: (1) fissural orientation, (2) posterior, and (3) anterior hilar orientation. The bedside technician performed real-time model alignment during these stages, deforming the iSR model as needed while observing the surgery's progress on a separate video monitor simultaneously. Surgical decision making was not influenced. AR visualization was performed through Intuitive's TilePro input. DL-based segmentation (minutes) and iSR model creation (seconds) times were documented.

## Results

**Figure 2:**
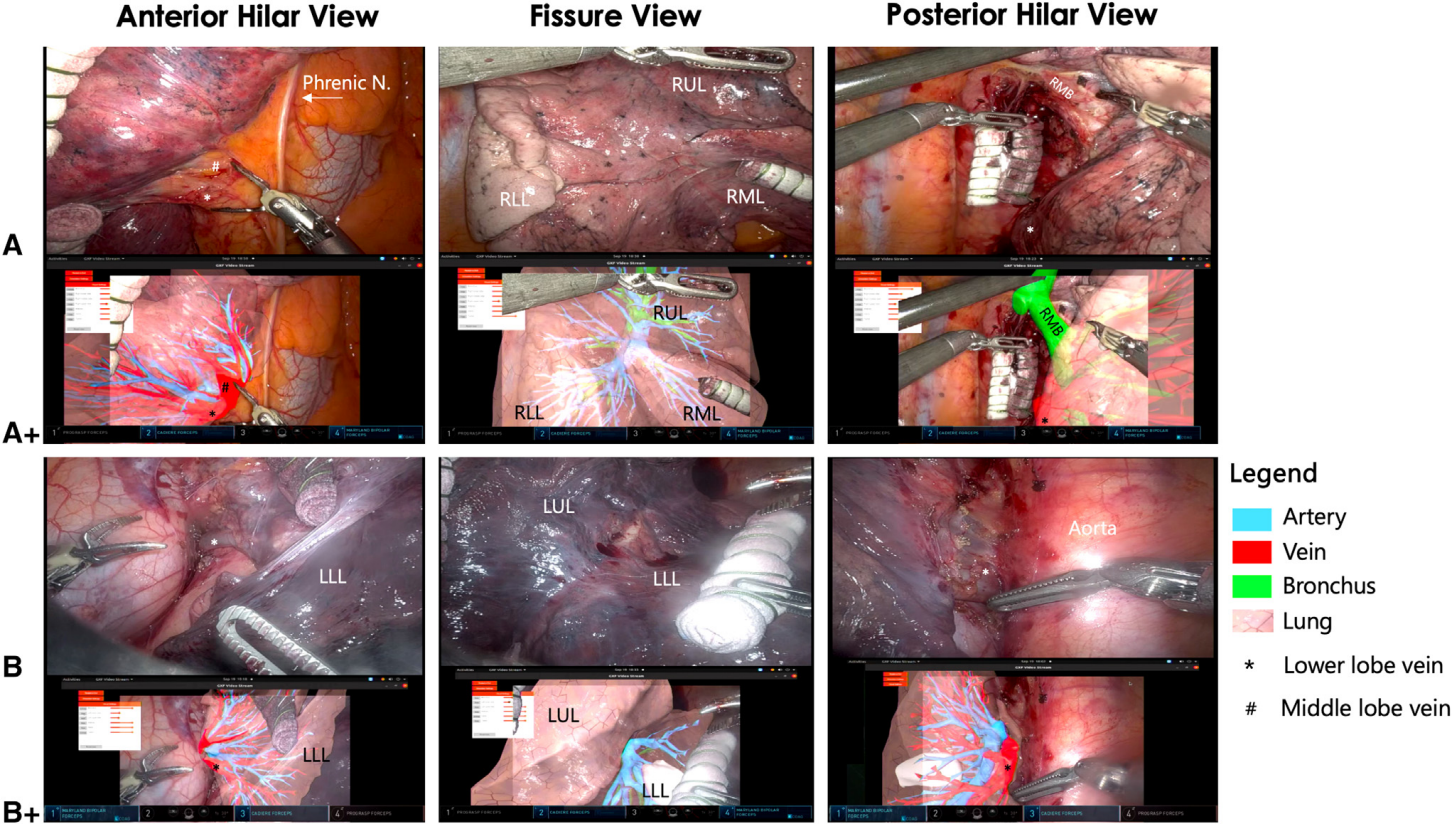
Intraoperative console images of robotic-assisted lung surgery enhanced with augmented reality. Intraoperative console screenshots of a right lower lobectomy (row A) and left lower lobectomy (row B) are presented, during 3 standard surgical phases of a robotic lower lobectomy. Both the surgical view (A and B, *top row*) and the Tilepro input, augmented with 3-dimensional projections of deformable simulated reality models, (row A+ and row B+) can be seen simultaneously in the surgeon's console view. *Phrenic N*, Phrenic nerve; *RUL*, right upper lobe; *RLL*, right lower lobe; *RML*, right middle lobe; *RMB*, right main bronchus; *LLL*, left lower lobe; *LUL*, left upper lobe.

Successful real-time implementation of the technological setup in Figure 1 was achieved. Both AR registration and dynamic tracking were performed effectively in both cases, as judged by the 2 console surgeons (Video 1). In both cases, successful AR superimposition of the iSR lung models during all 3 (standard) surgical phases was achieved (Figure 2). The 3D reconstructions were judged to be anatomically correct and realistic. Both lobectomies were successful, with minimal blood (20 mL) loss, and the postoperative course was uneventful. The patients were discharged on postoperative days 3 and 5, respectively. Table E1 summarizes the relevant technical results.

## Discussion

In this brief research article, we present for the first time the technological feasibility of an AI-based approach to enable AR (registration and dynamic tracking) for the purpose of RATS lung resections in 2 lower lobectomies. We believe that we have demonstrated solutions to some of the challenges associated with AR for robotic-assisted surgery in deformable organs (eg, lung tissue). 3D reconstructions have been shown to improve RATS; however, these models fail to account for the intraoperative altered lung shape resulting from lung desufflation, manual manipulation, and nonanatomical positioning. Here, we have demonstrated the use of deformable iSR models as a potential solution to this challenge. Even though we have shown the technological feasibility of AR-guided lung surgery, challenges remain with regards to the registration/dynamic tracking during surgery. Currently, the technology lacks the capability for virtual dissection, ligation, or stapling of structures and lung parenchyma. This limitation may present challenges when employing the technology for more intricate procedures like upper lobectomies or segmentectomies, where multiple structures are ligated, and the lung experiences extensive deformation as the result of parenchymal stapling. In addition, with the currently described method, it is not (yet) possible to perform automated registration or dynamic tracking of the tissue. A potential solution under exploration is the use of DL-based recognition of anatomical landmarks to facilitate automated landmark-based registration of the iSR-model. In addition to technological improvements, future clinical validation studies are needed to demonstrate the clinical benefits (eg, shorter operation times/learning curves) and applications (eg, localization of deeply located noduli) of AR-guided lung surgery.

## Conflict of Interest Statement

Dr Sadeghi is a co-inventor of PulmoVR. Q. Mank (part-time) and A. Tuzcu are MedicalVR (Nieuw-Vennep, The Netherlands) employees. P. De Backer, A. Mottrie, and J. Hofman are Orsi Academy (Melle, Belgium) employees. All other authors reported no conflicts of interest.

The *Journal* policy requires editors and reviewers to disclose conflicts of interest and to decline handling or reviewing manuscripts for which they may have a conflict of interest. The editors and reviewers of this article have no conflicts of interest.

## Figures and Tables

**Table E1:** Summary of technical characteristics

Case	DL-based segmentation of artery, s	DL-based segmentation of vein, s	DL-based segmentation of bronchus, s	DL-based segmentation of lobes, s	DL-based segmentation total, min:s	Rendering time iSR 3D model, s
Right lower lobectomy case (entire lung lobe segmentation)	373	365	59	40	13:57	18
Left lower Lobectomy (entire lung lobe segmentation)	302	309	59	40	11:50	13

Summarized are the times needed for the creation of the 3D and iSR models by the artificial intelligence- and finite element method–based algorithms. *DL*, Deep learning; *iSR*, interactive simulated reality; *3D*, 3-dimensional.

## Supplementary Data

Video 1 Real-time visualization of artificial intelligence–based augmented reality overlay during robotic-assisted lung resection. A representative intraoperative video of the “posterior orientation” surgical phase demonstrating the surgeon's view (*upper panel*) and the real-time augmented reality overlay (*lower panel*) of the virtual lung model showing the trachea, right main bronchus with the upper lobe bronchus division (and its segmental branches), the intermediate bronchus and the right pulmonary artery. Video available at: https://www.jtcvs.org/article/S2666-2507(24)00170-6/fulltext.
