# Supplementary figures and images for: Artificial intelligence–assisted augmented reality robotic lung surgery: Navigating the future of thoracic surgery
Source: JTCVS Tech. 2024 May 3;26:121–5. doi: 10.1016/j.xjtc.2024.04.011 (PMC11329169; doi:10.1016/j.xjtc.2024.04.011)

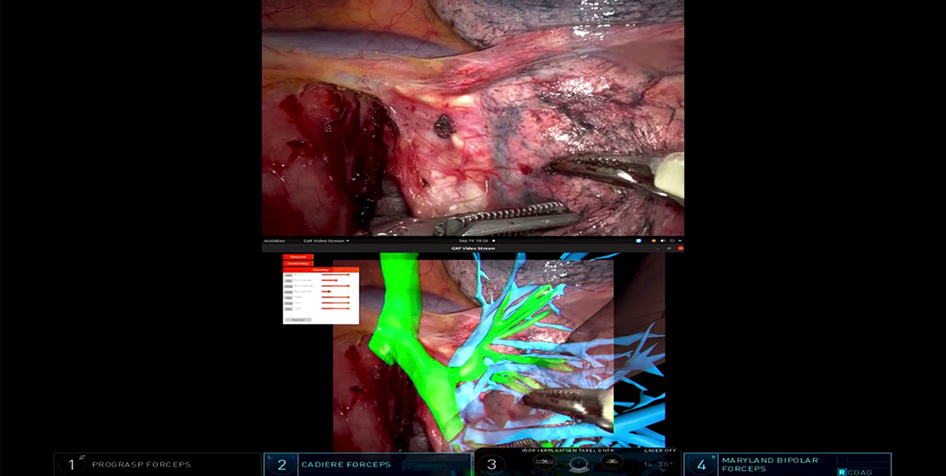

Supplement: Video 1 — Real-time visualization of artificial intelligence–based augmented reality overlay during robotic-assisted lung resection. A representative intraoperative video of the “posterior orientation” surgical phase demonstrating the surgeon's view (upper panel) and the real-time augmented reality overlay (lower panel) of the virtual lung model showing the trachea, right main bronchus with the upper lobe bronchus division (and its segmental branches), the intermediate bronchus and the right pulmonary artery. Video available at: https://www.jtcvs.org/article/S2666-2507(24)00170-6/fulltext. [file fx2.jpg]
